# Mental health problems in a regional population of Australian adolescents: association with socio-demographic characteristics

**DOI:** 10.1186/s13034-016-0120-9

**Published:** 2016-09-13

**Authors:** Julia Dray, Jenny Bowman, Megan Freund, Elizabeth Campbell, Rebecca K. Hodder, Christophe Lecathelinais, John Wiggers

**Affiliations:** 1 Hunter New England Population Health Research Group, Hunter New England Local Health District, Wallsend, NSW Australia; 2 Faculty of Science and IT, School of Psychology, University of Newcastle, University Drive, Callaghan, NSW Australia; 3 Faculty of Health and Medicine, School of Medicine and Public Health, University of Newcastle, University Drive, Callaghan, NSW Australia; 4 Hunter Medical Research Institute, New Lambton Heights, NSW Australia

**Keywords:** Mental health problems, SDQ, Adolescent, Socio-demographic characteristics

## Abstract

**Background:**

Population level data regarding the general mental health status, and the socio-demographic factors associated with the mental health status of adolescents in Australia aged 12–16 years is limited. This study assessed prevalence of mental health problems in a regional population of Australian students in Grades 7–10, and investigated associations between mental health problems and socio-demographic factors.

**Methods:**

A web-based survey was conducted in 21 secondary schools located in disadvantaged local government areas in one regional local health district of NSW Australia. Mental health problems were measured using the youth self-report Strengths and Difficulties Questionnaire (SDQ) total SDQ score and three subscale scores (internalising problems, externalising problems and prosocial behaviour). Associations between each SDQ outcome and student socio-demographic characteristics (age, gender, Aboriginal and/or Torres Strait Islander Status, remoteness of residential location and socio-economic disadvantage) were investigated.

**Results:**

Data are reported for 6793 students aged 12–16 years. Nineteen percent of participants scored in the ‘very high’ range for the total SDQ, 18.0 % for internalising problems, 11.3 % for externalising problems and 8.9 % for prosocial behaviour problems. Gender and Aboriginal status were associated with all four SDQ outcomes, while age was associated with two, excluding externalising problems and prosocial behaviour. Aboriginal adolescents scored higher for mental health problems than non-Aboriginal adolescents for all four SDQ outcomes. Females scored higher than males for total SDQ and internalising problems, with mean difference greatest at age 15. Males scored higher for externalising problems and lower for prosocial behaviour than females.

**Conclusions:**

The finding that mental health problems significantly varied by age, gender and Aboriginality may suggest a need for tailored interventions for groups of adolescents with highest levels of mental health problems.

*Trial Registration* ANZCTR ACTRN12611000606987. Registered 14/06/2011.

## Background

Globally, it is estimated that between 1.8 and 39.4 % of young people aged 0–16 years experience mental health problems [1], with such problems accounting for 15–30 % of disability adjusted life-years lost during the first three decades of life [2]. The wide range of prevalence estimates has been suggested to be attributable to differences between studies in the populations (including age groups studied), risk and protective factor characteristics of the samples, the measurement approaches and tools used [1, 3]. Further, such differences have been attributed to cultural contexts, where cultural background may impact on the expression and evaluation of symptoms of mental health problems and level of impairment [1, 3].

Population level studies of mental health problems are suggested to require standardised measurement tools that can be feasibly implemented on a large-scale [4]. In addition, tools that provide a measure of the general mental health status of participants rather than of specific diagnostic conditions, and that can be administered without extensive clinical knowledge, are recommended in describing the mental health of the adolescent population overall, and of particular groups within the adolescent population [5, 6].

Limited population level data have been reported regarding the mental health status of adolescents [7], with adolescence being defined as the second decade of life [8]. Where such data exist, there is considerable variability regarding the extent to which it meets the above best practice measurement recommendations for population level studies [3]. For example, a recent report regarding child and adolescent mental health data in 15 European countries found few to have data regarding the mental health status of adolescents that met such recommendations [6]. The report noted that existing population prevalence surveys differed in terms of the age ranges covered, the recency of data collection, the mental health problems assessed and the measurement instruments used, with most countries reporting the prevalence of specific mental health disorders and not of mental health status generally [6].

In contrast, systematic collection of population level adolescent mental health data has occurred in the United Kingdom through the National Survey of Mental Health of Children and Young People [5]. The most recent survey was undertaken in 2004 [5], with a follow-up study addressing age of onset and persistence conducted in 2007 [9]. Children and adolescents aged 5–16 years were assessed using a battery of items including the Development And Well-Being Assessment (DAWBA) tool [5]. Based on the DAWBA tool, the 2004 survey identified 10 % of young people aged 5–16 years to have a clinically diagnosed mental disorder, with prevalence being greater for: older children; males; some ethnic groups; and for adolescents with parents who were socio-economically disadvantaged [5]. The prevalence of clinically diagnosed mental disorders for adolescents aged 11–15 years was 12 % [5].

Similarly, in the United States of America, the National Health Interview Survey (NHIS; conducted since 1957) was adapted from 2001 to include the parent-report version of the Strengths and Difficulties Questionnaire (SDQ) [10], with some components of the SDQ being included in the survey annually until present. The SDQ is a standardised measure of mental health problems in children and adolescents, with established reliability and validity [11, 12]. From 2001 to 2007, the NHIS found 2 % of children and adolescents aged 4–17 years to have high scores on the brief version of the SDQ, with prevalence highest amongst older children (2.6 % for both adolescents 11–14 years and 15–17 years: 10). Additionally prevalence was found to be similar for males and females (2.3 and 2.1 % respectively), and to vary by race, language, ethnicity, family type, family income and type of health insurance [10].

In Australia, the collection of recent population level data regarding the general mental health status of adolescents has been limited, with a noted gap in such data particularly for young Australians aged 12–15 years [13]. The National Survey of Mental Health and Wellbeing has incorporated a child and adolescent component twice; in 1998 [14] and most recently in 2013–2014 [15]. In the recent administration, retitled the Young Minds Matter Survey [15] the prevalence of very high psychological distress, measured by the Kessler 10 (K10), and prevalence of mental health problems, measured by scores in the ‘abnormal’ range on the SDQ in adolescents aged 11–17 years, was indicated to be 13.3 and 10.2 % respectively. In another recent national survey, the Mission Australia Youth Survey (2013) the prevalence of probable serious mental illness in adolescents aged 15–19 years, measured using the Kessler 6 (K6), was estimated to be 21.2 % [16]. The authors could identify two further publications reporting population level prevalence data on general mental health problems for Australian adolescents collected since the year 2000, both undertaken in the state of Victoria [17, 18]. In the first, undertaken in 2001–2002 among a random sample of children and adolescents aged 7–17 years, prevalence of mental health problems, as measured by scores in the ‘abnormal’ range on the youth self-report SDQ, was reported to be 5.8 % [17]. In the second undertaken in 2009–2010, a larger state wide survey of adolescents aged 11–18 years, prevalence of very high psychological distress, as measured by the K6, was reported to be 13 % [18].

Three of the four recent Australian studies described above investigated mental health problems by gender and age although the findings were somewhat inconsistent: two reporting a higher prevalence for females [15, 16], and the other for males [17]; and similarly, two reporting limited variation in prevalence by age [16, 17], and the other a higher prevalence for older adolescents aged 16–17 years as compared to those aged 11–15 years [15]. Only one study, the more recent of the two conducted in Victoria, assessed differences in mental health status between rural and metropolitan areas, with no differences found [18]. Likewise only one study, one of the two national surveys, examined differences by Aboriginal status, reporting a higher prevalence of mental health problems among Aboriginal adolescents [16]. None of the four studies examined prevalence of mental health status by socio-economic disadvantage.

The aims of the present study were to (1) determine the prevalence of mental health problems in a regional sample of adolescents aged 12–16 years, attending secondary schools located in disadvantaged local government areas in one local health district of NSW, Australia, and (2) investigate associations between mental health problems and a range of socio-demographic characteristics (age, gender, Aboriginal status, remoteness of residential location and socio-economic disadvantage).

## Methods

### Study design and setting

A cross sectional survey was undertaken in a regional local health district of New South Wales, Australia, from August to November in 2011. The region covers an area of approximately 130,000 square km [19], and consists of a large metropolitan centre, regional centres, and rural and remote communities, with an estimated population of 115,000 adolescents aged from 10 to 19 years [20]. Relative to the state of NSW, the area has a lower index of socio-economic status [20, 21], a higher proportion of people residing outside metropolitan areas, and a higher proportion of the adolescent population (10–19 years) are Aboriginal (9.6 vs 5.3 % in NSW) [20]. The survey was conducted as part of a randomised controlled trial registered with the Australia and New Zealand Clinical Trials Register (Ref no. ACTRN12611000606987) details of which are described elsewhere [22].

### Ethics, consent and permissions

Ethics approval was obtained from: the Hunter New England Health Human Research Ethics Committee (Ref no. 09/11/18/4.01); The University of Newcastle Human Research Ethics Committee (Ref no. H-2010-0029); the Aboriginal Health and Medical Research Council (Ref no. 776/11); the New South Wales Department of Education and Training State Education Research Approval Process (Ref no. 2008118), and relevant Catholic Schools Offices.

Students with parental consent were invited to complete a self-report web-based survey within class time, supervised by school staff and members of the research team. Student verbal agreement to participate was required at the time of data collection.

### Sample and recruitment

#### Secondary schools

Schools were eligible to participate in the study if they: had a student population of at least 400 students; enrolments across Grades 7–10 (typically aged from 12 to 16 years); were co-educational; and located within a disadvantaged Local Government Area (school postcode in a Local Government Area with a score of <1000 on the Socio-Economic Indexes for Areas, SEIFA; 23). Boarding schools, central schools (catering for students aged 5–18 years), and special needs or selective schools were ineligible to participate. Forty-seven schools were eligible for participation in the trial, forty-four of which were randomly approached until a quota of 32 schools was achieved. Data for this study were collected from a sample of 21 such schools as these schools had a measure of mental health problems included in the student survey.

#### Student sample

All students enrolled in Grades 7–10 and aged 12–16 years were eligible to participate. Study information packs (an information letter for parents, a simplified study information letter for students, a consent form, and a reply paid envelope) were mailed to parents. Existing school communication channels were employed to promote student participation [24]. Non-responding parents were phoned by school-affiliated staff and asked to provide verbal consent or non-consent for their child to participate. For parents who provided verbal consent, a replacement study information pack was provided by mail.

Additional strategies were employed to support participation by Aboriginal students. Where possible and following approval by each school Principal, an Aboriginal member of the research team made contact with an Aboriginal staff member from each school. Additionally, the contact number of both a male and female Aboriginal member of the research team was provided in the study cover letter for parents to contact about the study. Finally, information relating to the study was presented to Aboriginal groups and services within the study area.

### Measures

#### Mental health problems

Mental health problems were assessed using the 25-item youth self-report version of the SDQ [11].The SDQ has been identified as one of the key measurement tools for use in Australian child and adolescent mental health services [25], a tool for which normative data exists for Australian school students aged 7–17 years [17]. The SDQ consists of five subscales: emotional symptoms; conduct problems; hyperactivity/inattention; peer relationship problems; and prosocial behaviour; with each subscale containing five items in the form of statements requiring a response via a three point Likert response scale: 0 (not true); 1 (somewhat true); or 2 (certainly true) [11].

As well as collecting data on the mental health of adolescents through the use of the SDQ, the survey included items regarding adolescent health behaviours such as substance use, physical activity, sexual health (Grade 10 students only), and bullying. The mean survey completion time was 22.6 min (SD: 10.2) with 90 % of students completing in 30 min or less and completion of the SDQ component taking approximately 5 min of the completion time. Aboriginal students answered additional survey questions, therefore the mean completion time for Aboriginal students was 23.9 min (SD: 7.93), with 90 % of Aboriginal students completing in 33 min or less.

#### Student characteristics

The survey contained items relating to student age, gender, Aboriginal and/or Torres Strait Islander status (‘Are you of Aboriginal or Torres Strait Islander origin?’: ‘Yes, Aboriginal origin’; ‘Yes, Torres Strait Islander origin’; ‘Yes, both Aboriginal and Torres Strait Islander origin’; ‘No’), and residential postcode.

#### Statistical analysis

All analyses were conducted using the statistical program SAS, Version 9.3 [26].

##### Student characteristics

Descriptive statistics were used to examine parental consent rates, student participation rates, and student demographic characteristics. Aboriginal and/or Torres Strait Islander status (hereafter referred to as Aboriginal) was based on student self-report during the survey. Participant residential postcodes were used to derive their socio-economic disadvantage score according to SEIFA; postcodes were classified into quintiles, where quintile 1 was the most disadvantaged and quintile 5 the least disadvantaged [23]. For the variable of socio-economic disadvantage, quintiles 4 and 5 were combined due to a small number of participants in quintile 5 (see Table 2). Data relating to remoteness of residential location were calculated from participants’ residential postcodes based on scores of the Accessibility/Remoteness Index of Australia (ARIA) and grouped into three categories: major city; inner regional; and outer regional/remote [27].

##### Mental health problems

Students that did not complete all 25 SDQ items were excluded from the analysis. An approach that reduces the five sub-scale structure of the SDQ to a three subscale structure has been recommended when using the SDQ in general population studies [28] and was employed in this study. Such an approach is reported to be valid [29] and to reduce measurement error [30]. The three-subscale structure involves the items from the emotional symptoms and peer relationship problems subscales being combined to form a single ‘internalising’ subscale (10 questions; possible score range: 0–20), and the items from the conduct problems and hyperactivity/inattention subscales being combined to form a single ‘externalising’ subscale (10 questions; possible score range: 0–20), with the third subscale ‘prosocial behaviour’ remaining unchanged (5 questions; possible range: 0–10).

Subscale scores were calculated by adding responses to each item within each subscale [28]. The total difficulties score (total SDQ; possible range: 0–40) was calculated by adding the scores of the internalising and externalising subscales only [29].

To report prevalence of mental health problems, recent recommendations from the authors of the SDQ regarding the labelling of SDQ score categories and adaptation of the categories from a three to fourfold categorisation was adopted [31]. Recommended cut points (see Table 1) were used to identify the proportions of students scoring in the following ranges: ‘close to average’, ‘slightly raised’, ‘high’, and ‘very high’, for each of the four SDQ scores [31, 32].

**Table 1:** Cut-points used to report score ranges for each SDQ outcome (cut points obtained from 31, and 32)

	Score ranges			
	Close to average	Slightly raised	High	Very high
Total SDQ	0–14	15–17	18–19	20–40
Internalising problems	0–6	7–8	9	10–20
Externalising problems	0–8	9–10	11–12	13–20
Prosocial behaviour	7–10	6	5	0–4

##### Investigating associations between mental health problems and socio-demographic characteristics

To investigate associations between student socio-demographic characteristics and mental health problems, scores for the total SDQ and the three subscales were treated as continuous variables. Higher scores indicated greater mental health problems for total SDQ, internalising and externalising SDQ scores; and fewer problems for the prosocial behaviour scale [29, 31]. Associations between each participant socio-demographic characteristic (age, gender, Aboriginal status, remoteness of residential location and socio-economic disadvantage) and each SDQ outcome (total, externalising, internalising, and prosocial behaviour scores) were investigated using linear mixed models (20 models). For each SDQ score, all socio-demographic variables with a *p* < 0.20 were eligible to enter a backwards stepwise process, whereby non-significant variables were removed until all remaining variables were significant at *p* < 0.01. All possible combinations of remaining variables were tested for interaction effects, in order to determine the four final linear mixed models. All models included a random effect for school, to account for clustering of responses within schools.

## Results

### Sample

Across the 21 schools, out of 12,134 eligible enrolled students, parental consent was granted for 9241 students (76.2 %), of whom 6879 completed the student survey (participation rate of students with parental consent 74.4 %). Hence the sample represents 56.7 % of the total enrolled student population. Participants who did not complete all the SDQ survey items were excluded from analysis (n = 86), leaving a final study sample of 6793 participants. Demographic characteristics of the sample are described in Table 2, illustrating comparability with the full school sample in the larger trial.

**Table 2:** Descriptive statistics of participating students demographics

Student demographic	Current sample (21 schools; n = 6793)		Full study sample (32 schools; n = 10,116)	
	n	%	n	%
Gender				
Male	3390	49.9	5061	50.0
Age				
12	829	12.2	1268	12.5
13	2008	29.6	2934	29.0
14	1799	26.5	2670	26.4
15	1484	21.8	2237	22.1
16	673	9.9	1007	10.0
Aboriginality				
Aboriginal and/or Torres Strait Islander	732	10.8	1144	11.3
Socioeconomic disadvantage^a^				
Quintile 1 (most disadvantaged)	725	10.7	1276	12.6
Quintile 2	2090	30.8	3201	31.7
Quintile 3	2781	41.0	4344	43.0
Quintile 4	1116	16.5	1211	12.0
Quintile 5 (least disadvantaged)	68	1.00	68	0.7
Remoteness (ARIA)^a^				
Major cities Australia	3311	48.8	4892	48.4
Inner regional Australia	2611	38.5	4119	40.8
Outer regional/remote Australia	860	12.7	1094	10.8

Relative to the state of NSW, both the current study sample and wider study region have a similar gender composition for the adolescent population (49.9, 51.5 and 51.5 % male; current sample, region and state respectively), however have a lower index of socio-economic status [20, 21], a higher proportion of people residing outside metropolitan areas, and a higher proportion of the adolescent population (10–19 years) are Aboriginal (10.8, 9.6 and 5.3 %; current sample, region and state respectively) [20]

^a^Sample size varied due to missing data

### Mental health problems

The proportion of participants scoring in the ‘close to average’, ‘slightly raised’, ‘high’ and ‘very high’ range for mental health problems is shown in Table 3. The prevalence of participants scoring ‘very high’ was 19.0 % for total SDQ score, 18.0 % for internalising problems, 11.3 % for externalising problems and 8.9 % for prosocial behaviour problems. A further 7.9, 6.3, 10.9 and 11.6 % had scores in the ‘high’ range for each of these outcomes respectively.

**Table 3:** Prevalence of scores in the ‘close to average’, ‘slightly raised’, ‘high’ and ‘very high’ range for total SDQ and three SDQ subscales

Score range	Outcome (N = 6973)			
	Total SDQ	Internalising	Externalising	Prosocial
	n (%)	n (%)	n (%)	n (%)
Close to average	4041 (59.5)	4074 (60.0)	4185 (61.6)	4400 (64.8)
Slightly raised	927 (13.6)	1074 (15.8)	1099 (16.2)	1001 (14.7)
High	533 (7.9)	425 (6.2)	742 (10.9)	786 (11.6)
Very high	1292 (19.0)	1220 (18.0)	767 (11.3)	606 (8.9)

### Associations between mental health problems and socio-demographic characteristics

Mean scores and standard deviations for total SDQ and each of the three subscales are reported for all participants and by socio-demographic groups in Table 4. Mean total SDQ for all participants was 13.43 (SD = 6.49), with mean scores of 5.98 (SD = 3.72) for the internalising subscale, 7.45 (SD = 3.95) for the externalising subscale, and 7.19 (SD = 1.97) for the prosocial behaviour subscale.

**Table 4:** Mean scores and standard deviations for total SDQ, internalising, externalising and prosocial SDQ subscales by socio-demographic factors

	Outcome				
		Total SDQ (0–40)	Internalising (0–20)	Externalising (0–20)	Prosocial (0–10)
	n	Mean (SD)	Mean (SD)	Mean (SD)	Mean (SD)
All	6793	13.43 (6.49)	5.98 (3.72)	7.45 (3.95)	7.19 (1.97)
Gender		*p* < 0.0001	*p* < 0.0001	*p* < 0.0001	*p* < 0.0001
Male	3390	12.96 (6.34)	5.31 (3.57)	7.64 (3.92)	6.72 (2.04)
Female	3403	13.90 (6.61)	6.64 (3.74)	7.25 (3.97)	7.66 (1.78)
Age		*p* < .01	*p* <0 .01	*p* = 0.06	*p* = 0.25
12	829	13.12 (6.48)	5.78 (3.61)	7.35 (4.02)	7.34 (1.93)
Male	360	13.31 (6.23)	5.39 (3.43)	7.91 (3.93)	6.94 (1.99)
Female	469	12.98 (6.66)	6.07 (3.73)	6.91 (4.03)	7.65 (1.82)
13	2008	13.15 (6.45)	5.79 (3.64)	7.36 (3.94)	7.20 (1.89)
Male	1024	13.03 (6.41)	5.42 (3.63)	7.61 (3.87)	6.77 (1.93)
Female	984	13.28 (6.49)	6.18 (3.61)	7.10 (4.01)	7.64 (1.75)
14	1799	13.52 (6.54)	5.97 (3.74)	7.54 (4.00)	7.15 (2.08)
Male	885	12.93 (6.41)	5.21 (3.55)	7.72 (4.05)	6.56 (2.18)
Female	914	14.09 (6.62)	6.72 (3.77)	7.38 (3.94)	7.71 (1.80)
15	1484	13.94 (6.46)	6.30 (3.82)	7.64 (3.85)	7.14 (1.97)
Male	736	12.79 (6.15)	5.19 (3.52)	7.60 (3.82)	6.68 (2.05)
Female	748	15.07 (6.56)	7.39 (3.78)	7.69 (3.89)	7.60 (1.77)
16	673	13.25 (6.53)	6.10 (3.74)	7.15 (3.95)	7.21 (1.94)
Male	385	12.83 (6.44)	5.44 (3.67)	7.39 (3.95)	6.85 (1.96)
Female	288	13.81 (6.62)	6.97 (3.66)	6.83 (3.94)	7.70 (1.80)
Aboriginality		*p* < 0.0001	*p* < 0.0001	*p* < 0.0001	*p* < 0.001
Aboriginal and/or Torres Strait Islander	732	15.41 (6.69)	6.70 (3.87)	8.71 (4.01)	6.90 (2.07)
Non-Aboriginal	6061	13.19 (6.43)	5.89 (3.69)	7.30 (3.92)	7.23 (1.95)
Socioeconomic Disadvantage (SED)^a^		*p* = 0.09	*p* = 0.17	*p* = 0.26	*p* = 0.43
Quintile 1 (most disadvantaged)	725	13.22 (6.69)	5.88 (3.76)	7.34 (4.04)	7.12 (1.96)
Quintile 2	2090	13.62 (6.41)	6.11 (3.67)	7.52 (3.93)	7.21 (1.94)
Quintile 3	2781	13.55 (6.48)	6.01 (3.75)	7.53 (3.94)	7.18 (2.00)
Quintile 4 and 5	1184	12.89 (6.50)	5.71 (3.68)	7.18 (3.91)	7.24 (1.96)
Quintile 4	1116	12.97 (6.49)	5.75 (3.65)	7.23 (3.93)	7.20 (1.97)
Quintile 5 (least disadvantaged)	68	11.57 (6.60)	5.15 (4.17)	6.43 (3.52)	7.91 (1.70)
Remoteness (ARIA)		*p* = 0.63	*p* = 0.25	*p* = 0.97	*p* = 0.29
Major cities Australia	3311	13.50 (6.55)	6.06 (3.77)	7.45 (3.96)	7.24 (1.97)
Inner regional Australia	2611	13.35 (6.46)	5.91 (3.66)	7.44 (3.97)	7.18 (1.97)
Outer regional/remote Australia	860	13.34 (6.35)	5.86 (3.65)	7.48 (3.86)	7.00 (1.95)

^a^For all statistical analyses quintiles 4 and 5 were combined due to a small sample distribution of participants and schools in quintile 5

The results of the 20 models testing for associations between each socio-demographic characteristic and SDQ score are shown in Table 4. Results of the final four linear mixed models for each SDQ score are shown in Table 5.

**Table 5:** Results of final linear mixed models of socio-demographics by mental health problems

		Outcome		
	Total SDQ (0–40)	Internalising (0–20)	Externalising (0–20)	Prosocial (0–10)
	Mean difference (95 % CI)	Mean difference (95 % CI)	Mean difference (95 % CI)	Mean difference (95 % CI)
Gender	*p* < 0.0001	*p* < 0.0001	*p* < 0.0001	*p* < 0.0001
Female	0.95 (−0.09 to1.98)	1.51 (0.92 to 2.10)	−0.39 (−0.58 to −0.19)	0.93 (0.83 to 1.02)
Male	–	–	–	–
Age	*p* < .01	*p* < .001	n.s.	n.s.
12	0.45 (−0.48 to 1.38)	−0.05 (−0.58 to 0.48)		
13	0.10 (−0.66 to 0.86)	−0.06 (−0.50 to 0.37)		
14	0.04 (−0.73 to 0.82)	−0.25 (−0.69 to 0.19)		
15	−0.15 (−0.94 to 0.65)	−0.29 (−0.74 to 0.16)		
16	–	–		
Age × gender	*p* < .0001	*p* < .0001	n.s.	n.s.
12 × female	−1.31 (−2.64 to −0.02)	−0.85 (−1.60 to −0.10)		
*Female*	−*0.36 (*−*1.25 to 0.53)*	*0.66 (0.16 to 1.16)*		
13 × female	−0.65 (−1.79 to 0.48)	−0.73 (−1.37 to −0.08)		
*Female*	*0.29 (*−*0.27 to 0.86)*	*0.78 (0.46 to 1.10)*		
14 × female	0.22 (−0.94 to 1.37)	−0.01 (−0.66 to 0.65)		
*Female*	*1.16 (0.57 to 1.76)*	*1.50 (1.16 to 1.84)*		
15 × female	1.33 (0.14 to 2.52)	0.68 (0.01 to 1.35)		
*Female*	*2.28 (1.62 to 2.94)*	*2.19 (1.82 to 2.57)*		
16 × Female	–	–		
*Female*	*0.95 (*−*0.04 to 1.93)*	*1.51 (0.95–2.07)*		
Aboriginality	*p* < 0.0001	*p* < 0.0001	*p* < 0.0001	*p* < 0.001
Aboriginal and/or Torres Strait Islander	2.02 (1.49 to 2.55)	0.70 (0.40 to 1.00)	1.33 (1.01 to 1.66)	−0.27 (−0.43 to −0.12)
Non-Aboriginal	–	–	–	–

For all analyses in table a statistical significance level of p ≤ 0.05 was assumed. Non-significant associations are indicated in the table using n.s. For n relating to all subscales please refer to Table 4

From the linear mixed models analyses, total SDQ score was associated with Aboriginal status, age and gender (see Table 5). Aboriginal students scored higher for mental health problems than non-Aboriginal students (β = 2.02, 95 % CI 1.49–2.55). There was a significant interaction between age and gender. Females scored higher for mental health problems than males for students aged 14 years (β = 1.16, 95 % CI 0.57–1.76), and 15 years (β = 2.28, 95 % CI 1.62–2.94), with mean difference greatest at 15 years; there was no significant gender difference for students aged 12 years (β = −0.36, 95 % CI −1.25 to 0.53), 13 years (β = 0.29, 95 % CI −0.27 to 0.86), and 16 years (β = 0.95, 95 % CI −0.04 to 1.93).

Internalising problems was associated with Aboriginal status, age and gender. Aboriginal students scored higher for internalising problems than non-Aboriginal students (β = 0.70, 95 % CI 0.40–1.00). There was a significant interaction between age and gender. Females scored higher for internalising problems than males for all age groups, with mean difference varying by age and greatest at age 15: 12 years (β = 0.66, 95 % CI 0.16–1.16), 13 years (β = 0.78, 95 % CI 0.46–1.10), 14 years (β = 1.50, 95 % CI 1.16–1.84), 15 years (β = 2.19, 95 % CI 1.82–2.57) and 16 years (β = 1.51, 95 % CI 0.95–2.07).

Externalising problems was associated with Aboriginal status and gender. Aboriginal students scored higher for externalising problems than non-Aboriginal students (β = 1.33, 95 % CI 1.01–1.66), and females scored lower for externalising problems than males (β = −0.39, 95 % CI −0.58 to −0.19). No significant interactions were found.

Prosocial behaviour was associated with Aboriginal status and gender. Aboriginal students scored lower for prosocial behaviour than non-Aboriginal students (β = −0.27, 95 % CI −0.43 to −0.12) and females scored higher for prosocial behaviour than males (β = 0.93, 95 % CI 0.83–1.02). No significant interactions were found.

Using linear mixed models, ad hoc analyses were conducted to further explore the pattern of results for Aboriginal and non-Aboriginal students. The analyses examined whether the association between Aboriginality and SDQ score held for the four component subscales of the broader internalising problems score (emotional symptoms, and peer relationship problems) and externalising problems score (conduct problems and hyperactivity/inattention). Aboriginal students scored higher than non-Aboriginal students across all component subscales (emotional symptoms *p* < 0.01, peer relationship problems *p* < 0.0001, conduct problems *p* < 0.0001, and hyperactivity/inattention *p* < 0.0001).

## Discussion

This study aimed to examine both the prevalence of, and a range of possible socio-demographic characteristics associated with mental health problems in a regional population of adolescents aged 12–16 years, attending secondary schools located in disadvantaged local government areas in one local health district in NSW, Australia. The results indicated nearly one-fifth (19 %) of the sampled adolescents scored in the ‘very high’ range for mental health problems overall, and slightly more than one quarter scored ‘high’ or ‘very high’ combined (27 %). Aboriginal students consistently scored higher for mental health problems for all outcome measures than non-Aboriginal students. Gender was associated with all outcome measures, with females scoring higher for total and internalising problems, and males scoring higher for externalising and lower for prosocial behaviour. Such findings may suggest a need for strategies to prevent and respond to mental health problems among young adolescents, particularly those with higher levels of mental health problems.

The finding that 19 % of students in the present sample scored ‘very high’ for mental health problems contrasts somewhat with two other surveys in Australia utilising the same measurement tool. A study of Victorian secondary school students aged 7–17 years conducted in 2001–2002 found that 5.8 % of Victorian school students were classified as ‘abnormal’, with this classification being equivalent to the ‘very high’ score range used in the present study [17]. Likewise, for total SDQ, Mellor et al. [17] reported a mean score of 8.9 for students aged 11–17 years, compared to a mean score of 13.4 in the current study for students aged 12–16 years. The most recent national survey conducted in 2013–2014 found 10.2 % of adolescents aged 11–17 years to fall into the ‘abnormal’ score range [15]; somewhat higher than the finding of Mellor [17] (5.8 %), but not as high as the prevalence of scores in the ‘very high’ range indicated in the current study (19 %).

A number of possible explanations may account for the different findings between these studies: an increase over time in the prevalence of mental health problems among adolescents; differences in the ages of students included in each study (7–17 years for Mellor, 11–17 years for Lawrence et al., and 12–16 years for the present study); differences in methods of administration, such as the use of online survey completion in the present study; and the focus of the present study on schools in disadvantaged local government areas within one local health district.

Aboriginal students were consistently found to score higher across all four SDQ outcomes, and also when compared on the smaller sub-scales. This finding aligns with previous studies indicating a higher prevalence of mental health difficulties among Aboriginal people generally [33] and among Aboriginal adolescents in particular [16, 34, 35]. Inequitable health outcomes are experienced by Aboriginal and/or Torres Strait Islander peoples for many health conditions, both physical and mental [36]. The markedly poorer health status of Aboriginal and/or Torres Strait Islander peoples has been attributed to a number of factors including, dispossession from land, government policies (e.g. stolen generation), experience of individual and institutional racism, and a lack of adequate access to education, housing and employment, and appropriate physical and mental health care services [37], similar to the health of other Indigenous peoples internationally [38]. It is important to consider how the above disadvantage and trans-generational trauma and loss has impacted on the social and emotional wellbeing of Aboriginal people including Aboriginal young people. However, it is equally as important to highlight resilience and strengths within Aboriginal individuals and communities including strong family and interpersonal relationships, maintenance of a unique cultural identity and connection, and the development of coping skills [37].

The finding of the present study that a greater proportion of female than male adolescents scored higher on the total SDQ score is consistent with results of the recent national study by Lawrence et al. [15], although not with the findings of Mellor [17], which found males scored higher in a sample of Victorian adolescents. The finding that female adolescents scored higher for internalising problems than males is consistent with both the previous studies utilising the SDQ in Australian samples, which indicate a higher prevalence of problems such as emotional symptoms for females compared to males [15, 17]. Likewise, the finding that male adolescents scored higher for externalising problems, is consistent with both previous studies which found males to have a greater prevalence of problems such as conduct problems and hyperactivity [15, 17]. For prosocial behaviour, the finding that females scored higher than males is consistent with the only other study reporting prevalence of prosocial behaviour problems for this age group in a sample of Australian adolescents [17]. Internationally, research utilising both the SDQ [39] and a range of other measures [40] also provides support for such differences in prevalence of internalising, externalising and prosocial behaviour problems by gender. Finally, the interaction results for the total SDQ and internalising problem scores in the present study, may suggest further investigation is required to fully understand gender and age differences in mental health problems in adolescents residing in the study region.

In accordance with previous research in Australia, the present study found no significant variation in the mental health of young people by socio-economic status and geographic location of residence [13, 18, 41]. Such findings are in contrast to international research indicating variation in the mental health status of adolescents by socio-economic status, with poorer mental health being evident for adolescents of lower socio-economic status [4, 42]. This differential may be explained by the recruitment in this study of students from schools in socio-economically disadvantaged areas or the use of an aggregate area-based measure of socio-economic disadvantage, not an individual-based measure. The finding of no differences in outcomes by geographic location of residence may also be attributable in part to the study being conducted largely in regional and rural areas, and thus being less representative of adolescents residing in metropolitan regions.

The findings of significant variation in mental health problems between groups of adolescents strengthen the need for the establishment of normative data for mental health problems in adolescents to be developed for Aboriginal and non-Aboriginal Australians, as well as for specific age and gender groups [17] as addressed in this study. The SDQ provides a basis for achieving this given the availability of a validated youth focused version of the tool and the existence of recommendations for the use of the SDQ in Australian child and adolescent mental health services [25]. However, fundamental differences in what the concept of mental health means for non-Aboriginal and Aboriginal people [43], may limit the appropriateness of the SDQ for Aboriginal young people. The term ‘social and emotional well-being’ has been used to describe the mental health of Aboriginal people, as it is a broad holistic term representing mental health as incorporating not only individual factors but additionally including wider factors such as cultural identification, spirituality and the community [35, 44]. The SDQ has been developed and validated with non-Aboriginal people and hence may not reflect the Aboriginal perspective of mental health. Three studies have assessed the appropriateness of the carer-report version of the SDQ with Aboriginal young people [45–47]. Whilst each of these studies suggests the SDQ to be, to an extent, an acceptable tool for the measurement of the mental health of Aboriginal people, all encourage further development of the tool to improve cultural appropriateness and clarity [45–47].

In addition to the limitations of the SDQ as a measure of the mental health of Aboriginal young people, interpretation of the study findings should be considered in light of a number of its design and methodological characteristics. First, the study was conducted using the self-report version of the SDQ. Previous research has suggested that exclusive reliance on adolescent self-report may result in under-reporting of mental health problems [11]. As a consequence, the observed prevalence of mental health problems may be an underestimate. Second, non-response bias is a common limitation of school-based research particularly due to absenteeism, refusals, and the additional need to obtain parental consent [48]. Thus whilst the parental consent rate and participation rate among students with parental consent were relatively high (76.2 and 74.4 % respectively), concerns remain about loss of ‘high-risk’ youth and subsequent possible underreporting of the prevalence of mental health problems in this group. Third, a number of factors may have influenced generalisability of the study findings. The data was obtained from baseline assessment for a larger intervention trial and SDQ data could only be obtained from 21 of 32 schools randomly selected for the larger trial, however student demographic characteristics are comparable to the full trial sample [22]. Additionally, the study was conducted in a single region within one Australian state. However, characteristics of the current sample are similar to that of the region in which the study was conducted in terms of socio-economic disadvantage, remoteness of residential location, gender and Aboriginality [49], supporting the demographic composition of the current sample as representative of the study region. In contrast, relative to the state of NSW, both the current sample and study region has a lower index of socio-economic status [20, 21], and a higher proportion of the adolescent population are Aboriginal [20]. Similarly, relative to the total population of young people in Australia the present study had a larger proportion of Aboriginal students and students from outside metropolitan areas [13, 50].

## Conclusions

In conclusion, the findings of the present study may suggest a need for tailored interventions for groups of adolescents with highest level of mental health problems. The current findings reinforce results of previous research [15, 17] in suggesting a need to target overall and internalising mental health problems in female students; and externalising problems and pro-social behaviour in males. Additionally, there remains a clear need for the development and validation of culturally appropriate measures of mental health for use with Aboriginal young people. Culturally appropriate measures would enable a more accurate indication of level of social and emotional health in Aboriginal adolescents, and better inform the need for any additional support. This will require active collaboration with Aboriginal community representatives and participation of Aboriginal researchers, to develop measurement tools and research methodology fully representative of factors considered as key indicators of the holistic concept of Aboriginal social and emotional wellbeing [51, 52].

## Abbreviations

ANZCTR: Australian New Zealand Clinical Trials Registry
DAWBA: Development and Well-Being Assessment Tool
NHIS: National Health Interview Survey
SDQ: Strengths and Difficulties Questionnaire
K6: Kessler Psychological Distress Scale (6-item version)
K10: Kessler Psychological Distress Scale (10-item version)
SEIFA: Socio-Economic Indexes for Areas
ARIA: Accessibility/Remoteness Index of Australia
